# Intracutaneous injection of autologous serum with specific IgE against penicillin followed by intracutaneous test with penicillin, does not elicit a positive intracutaneous test

**DOI:** 10.1186/2045-7022-5-S1-O10

**Published:** 2015-03-11

**Authors:** Line Kring Tannert, Charlotte Gotthard Moertz, Sidsel Falkencrone, Per Stahl Skov, Carsten Bindslev-Jensen

**Affiliations:** 1 Odense University Hospital, Allergy Centre, Department of Dermatology, Odense, Denmark

## Background

In patients allergic to penicillin only a minority of those who have specific IgE against penicillin also have a positive intracutaneous test (ICT) to penicillin and *vice versa.* This is in contrast to findings in patients with allergy to classical type 1 allergens, e.g. grass or peanuts. Besides that, our investigations indicates that histamine is only released in the minority of positive intracutaneous tests. The reason for this is not known. A hypothesis could be that the specific IgE against penicillin measured in serum is not present in the skin. The aim of this project is to investigate, if you by priming the skin with autologous serum in patients with specific IgE to penicillin subsequently are able to develop a positive ICT upon testing.

## Method

The patients are intradermally injected with autologous serum, 0,1 ml, in 3 different places on the volar forearm on day one.

On day two, cutaneous microdialysis is performed at the 3 primed sites and on two additional sites. Forwith microdialysis, semipermeable fibers with an inner diameter of 0,2 mm are inserted into the dermis and subsequently perfused with saline at a rate of 3 µl/min. During the experiment, ICT with penicillin is performed 1 mm away from the fiber both in the primed and not-primed areas. Histamine concentrations in the eluate are subsequently measured by fluorescence spectroscopy. ICT with saline and codeine served as negative and positive control in both primed and non-primed skin.

## Results

So far 8 patients have been investigated. All have measurable levels of IgE at the time of microdialysis. 6/8 were primed with serum obtained earlier where IgE levels were higher than the actual. On the day of the experiment 2/8 had a positive ICT in not-primed skin, the rest were negative. ICT performed in the primed areas did not result in any further positive ICTs. In the positive ICTs, no histamine was detected. All patients released significant amounts of histamine upon ICT with codeine.

## Conclusion

Our data do not support the hypothesis that the presence of IgE against penicillin and lack of a positive ICT is due to lack of IgE in the skin. Possibly, there may be both functional and non-functional IgE, or a positive ICT is not IgE mediated.

